# Occupational Heat Stress Among Migrant and Ethnic Minority Outdoor Workers: A Scoping Review

**DOI:** 10.1007/s40572-025-00481-y

**Published:** 2025-03-24

**Authors:** Lena van Selm, Sarah Williams, Francesca de’Donato, Erica Briones-Vozmediano, Jan Stratil, Gaby Sroczynski, Cathryn Tonne, Manuela De Sario, Ana Requena-Méndez

**Affiliations:** 1 Barcelona Institute for Global Health (ISGlobal), Barcelona, Spain; 2 Facultat de Medicina i Ciències de la Salut, Universitat de Barcelona (UB), Barcelona, Spain; 3 Department of Epidemiology, Lazio Regional Health Service/ASL Roma 1, Rome, Italy; 4 Department and Faculty of Nursing and Physiotherapy, University of Lleida, Lleida, Spain; 5 Research Group in Healthcare (GRECS), Biomedical Research Institute (IRB) Lleida, Lleida, Spain; 6 UMIT TIROL – University for Health Sciences and Technology, Hall in Tirol, Austria; 7 Universitat Pompeu Fabra (UPF), Barcelona, Spain; 8 CIBER Epidemiología y Salud Pública (CIBERESP), Madrid, Spain; 9 Department of Medicine Solna, Karolinska Institutet, Stockholm, Sweden; 10 Centro de Investigación Biomédica en Red de Enfermedades Infecciosas, CIBERINFEC, ISCIII - CIBER de Enfermedades Infecciosas, Instituto de Salud Carlos III, Madrid, Spain; 11 Consolidated Research Group in Society, Health, Education and Culture (GESEC), Lleida, Spain

**Keywords:** Heat Stress Disorders, Occupational Exposure, Occupational Health, Outdoor Workers, Transients and Migrants, Ethnic and Racial Minorities

## Abstract

**Purpose of review:**

Migrant and ethnic minority (MEM) outdoor workers might be at increased risk for heat-related illnesses (HRI), due to environmental exposures, heavy physical work, limited control over workplace conditions and language and cultural barriers. This review aims to synthesize the literature on health impacts of occupational heat exposure among MEM outdoor workers, including risk factors, heat-related perception and behaviour and healthcare utilization.

**Recent findings:**

Seventy-six publications were included. Most were conducted in the US, where the weighted prevalence for at least one HRI symptom was 48.8%. These numbers were higher in most non-US countries. On average, in the US, 60.9% reported being concerned about heat and 60.4% having had HRI training. Many workers reported drinking more water when hot (91.7%) and feeling comfortable taking water breaks (92%) while fewer reported acclimatizing at the start of the season (43.7%) or changing working hours (34%) or activities (32.2%) due to heat. Qualitative studies reported reasons for working faster with less breaks, including fear of losing work and earning more when getting paid by the piece. Data on access to healthcare was limited.

**Summary:**

While research advances on MEM workers´ heat-related vulnerability, risk factors and healthcare utilization, there is a need to strengthen prevention efforts to reduce the burden of heat in this population.

**Supplementary Information:**

The online version contains supplementary material available at 10.1007/s40572-025-00481-y.

## Introduction

Worldwide, 1.6 billion people were estimated to be formally engaged in outdoor occupations in 2022 [1]. Outdoor workers are at increased risk of occupational heat stress due to environmental exposures, including temperature, humidity, and direct sun exposure [2]. Heat stress refers to the accumulated heat exceeding the level that the human body can tolerate without physical impairment [3]. Heat stress can progress to heat-related illnesses (HRI) ranging from dizziness, heat strain or heat exhaustion to more severe forms such as heat stroke [4]. In addition, occupational heat stress decreases productivity, increases the rate of occupational injuries and can increase the risk of hospitalizations and death due to worsen pre-existing health conditions [2, 5–9]. In the long term, heat stress may progressively impair kidney function by leading to the development of chronic kidney disease [10].

In addition to environmental exposure, metabolic heat generation as a result of heavy physical work exacerbates the risk of heat stress for outdoor workers in certain industries, including construction and agriculture [8]. Personal characteristics, such as low physical fitness, lower skill levels, high or very low body mass index, higher age, pre-existing health conditions and the use of certain medications, drugs, or alcohol can further increase vulnerability to heat [11–13].

Migrants constitute a significant proportion of agricultural workers. In 2015, 16.7 million migrants were working in the agricultural sector globally and, in 2019, 7.1% of all global migrant workers were working in agriculture [14, 15]. In the US, between 2015–2018 over 75% of all farmworkers were migrants, almost exclusively from Central and South America [16]. In Europe, no reliable data are available but 26% of seasonal workers are estimated to be migrants (working up to nine months per year). However, this does not include migrants with permanent contracts nor migrants working without a contract [17, 18]. In addition, outdoor workers often belong to ethnic minorities, such as crop workers in the US, where 75% identify as Hispanic [19]. Ethnic minorities face similar societal and health challenges and disadvantages compared to migrant workers and in US studies on Latino / Hispanic workers, ethnicity and migration status are often conflated and used interchangeably [20–22]. Several factors such as irregular migratory status, low education levels and high economic needs can force migrant and ethnic minorities (MEM) to accept low-paid jobs under unfavorable conditions that could negatively impact their health [23]. In addition, limited control over workplace conditions and language and cultural barriers could further increase the risk of occupational injuries and diseases, including those related to heat [4, 24].

Although the adverse health effects of heat stress on outdoor workers have been well reported in the literature, a review focusing on heat stress among outdoor MEM workers is lacking. The aim of this review is to synthesize the literature on heat stress among outdoor MEM workers. A scoping review approach was chosen to be able to explore a broad range of themes related to occupational heat exposure among outdoor MEM workers, including: 1) prevalence of heat-related health outcomes, 2) risk- and protective factors for heat-related health outcomes, 3) knowledge, perception and behaviour related to occupational heat stress and 4) healthcare utilization and access to healthcare for health-related illnesses and injuries. Where possible comparisons are made with outcomes for non-MEM outdoor workers.

## Methods

We carried out a scoping review designed and reported complying with the Preferred Reporting Items for Systematic Review and Meta-analysis Protocols (PRISMA) guidelines [25]. A protocol was registered on Open Science Framework (OSF) (DOI 10.17605/OSF.IO/3XS4R).

### Search Strategy

Five electronic databases were searched on 20 November 2023: PubMed, Embase, Web of Science, CINAHL and PsycInfo. Studies in English and Spanish were included from any year of publication. In February 2024 the grey literature databases OpenGrey and Climate Change and Human Health Literature Portal were searched as well as the websites of the World Health Organization (WHO), International Organization for Migration (IOM), International Labour organization (ILO), Food and Agriculture Organization (FAO), and World Bank for relevant reports. Finally, the references of all papers were screened in three rounds of backwards- and one round of forward citation checking. To define the pillars of the search strategy we used the PECO approach, in which the Population, Exposure, Comparator and Outcomes are defined [26]. A broad search strategy was applied, combining two elements of PECO (Population: migrants and ethnic minorities outdoor workers; Exposure: heat) by using free text word and controlled vocabulary terms. Comparison and outcomes were applied during the assessment for eligibility and are explained below. Supplementary Table 1 includes the full search strategy.

### Eligibility Criteria

Experimental and observational quantitative studies and qualitative studies were considered eligible for inclusion. Conference proceedings, laboratory studies, modeling studies, case studies and reviews and meta-analyses were excluded. In addition, editorials, letters, books, consensus statements, or opinions were excluded.

Studies focusing on MEM outdoor workers were included. The IOM definition of migrant was used covering both international (migrated from a foreign country to country were working) and internal (migrated within the country of residence to another region for work) migrant workers [27]. Ethnic minorities were defined as differing from the majority ethnic group (“the social group a person belongs to, and either identifies with or is identified with by others, as a result of a mix of cultural and other factors including language, diet, religion, ancestry, and physical features traditionally associated with race”[28]). For practicality, if in studies at least 85% of the total sample consisted of MEM workers, the study was included. When disaggregated data were presented for MEM workers, only these data were included. Outdoor workers were defined by the occupational sector they work in, including categories that predominantly consist of outdoor tasks. According to Licker and colleagues, outdoor sectors were: protective service; buildings and grounds cleaning and maintenance; farming, fishing, and forestry; construction and extraction; installation, maintenance, and repair; transportation; and materials moving [29].

#### Study Outcomes

Studies with the following outcomes were included: a) health impacts of occupational heat exposure (physiological function, nonspecific signs and symptoms, heat-related symptoms or illnesses, kidney function, acute kidney injury (AKI) and chronic kidney disease (CKD), b) risk- and protective factors for heat-related health outcomes associated to a higher or lower frequency of symptoms, c) knowledge, perception and behaviour related to occupational heat exposure d) healthcare utilization and access to healthcare for health-related illnesses and injuries. Both self-reported and objectively measures outcomes were included.

#### Comparators/Controls

Both studies involving any type of comparison (MEM vs non-MEM populations or different MEM subgroups) and those with no comparator were included.

### Study Selection

Duplicates were removed from search results using the reference manager program Endnote version 21. Two reviewers (LvS and SW) independently screened first the titles and abstracts and later the full texts of all identified records using Rayyan according to predefined inclusion criteria [30]. Any disagreement was resolved by discussion between the two reviewers, with the involvement of a third reviewer (MDS) when necessary. Studies that did not meet the inclusion criteria were excluded and the reasons for exclusion were documented.

### Data Extraction

One reviewer extracted relevant data using a pre-defined extraction sheet and another double checked (LvS and SW). Extracted data included study characteristics (geographical location, year of publication, duration, and type of study) and quantitative or qualitative results for the heat-related outcomes of interest.

### Data Analysis

Due to heterogeneity in methods and outcomes, results were summarized in a narrative way. Where possible, prevalence estimates for the study outcomes were pooled across studies using weighted averages and confidence intervals calculated based on binomial distribution (Waller 1994) [31]. For qualitative studies, a thematic synthesis of results was conducted based on Thomas and Harden [32]. For each study outcome, direct participant quotes and author interpretations from qualitative studies were integrated in the narrative synthesis as critical interpretative perspective of quantitative results. Qualitative evidence summary is provided in the results and further details are provided in the Supplementary materials.

## Results

**Fig. 1:**
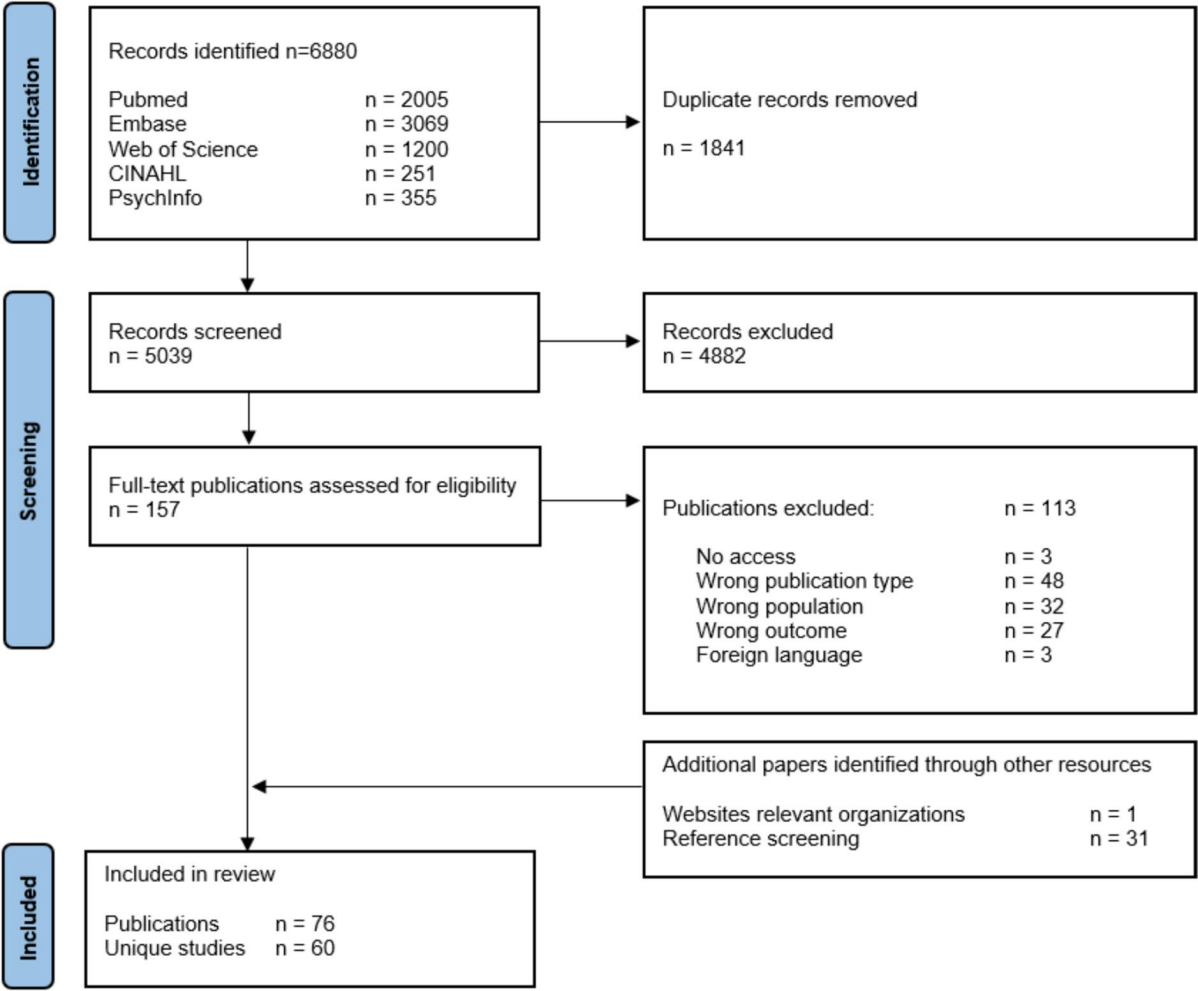
PRISMA flow diagram of included records and studies

**Fig. 2:**
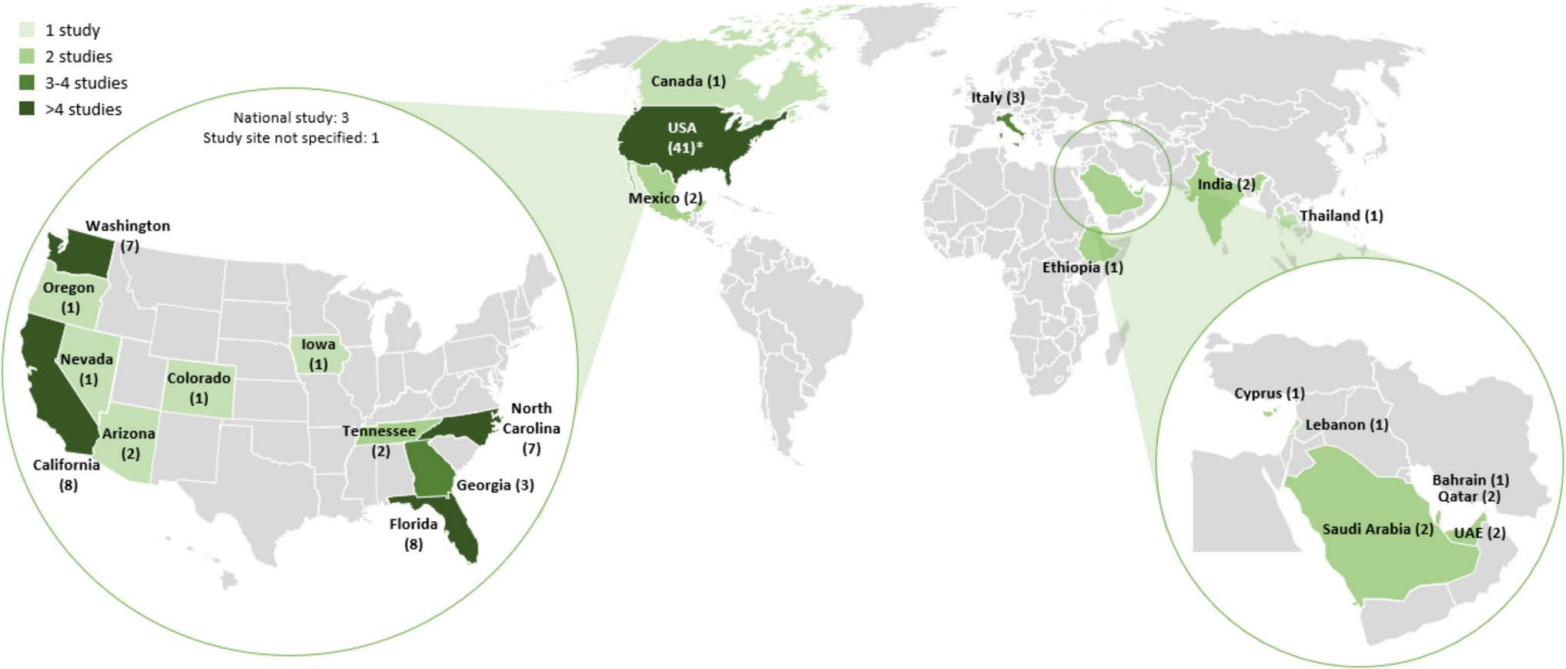
Overview of countries included in this review. Some studies have been conducted in multiple states, therefore the sum of studies per state exceeds the total number of studies | UAE: United Arab Emirates, USA: United States of America

A total of 5039 unique publications were identified searching the databases. Of these, 157 full texts were assessed, supplemented by 32 full texts identified via other sources, resulting in a total of 189 publications. Based on inclusion criteria, 113 publications were excluded, leaving only 76 for inclusion, presenting data from 60 distinct studies (Fig. 1).The included studies comprised 48 quantitative studies, 8 qualitative studies and 4 mixed-methods studies. Quantitative and mixed-methods study designs included cross-sectional studies (*n* = 42), case–control studies (*n* = 7) and cohort studies (*n* = 3). Most studies were conducted in the US (*n* = 41) or the Middle East (*n* = 8) (Fig. 2).

Table 1 shows an overview of the included papers and studies. Forty-one studies were conducted among farmworkers, mainly crop workers but also some nursery and fernery workers, seven among construction workers, one study included workers from both the agriculture and construction sector, whereas eleven included other, undefined or mixed groups of outdoor workers. Only eight studies included a definition of the term “migrant worker”, all of them referring to workers who change their place of residence for work. Studies included international migrants (*n* = 37), internal migrants (*n* = 11), or both international and internal (*n* = 3). Nine studies did not specify the migrant type or included ethnic minorities. Only studies from the US and Canada included populations belonging to an ethnic minority group and explicitly mentioned that participants were Latinos / Hispanics (*n* = 36).

**Table 1:** overview of included studies and papers

#	Reference	Country	Occupation	Design	MEM type †			Scoping review aim				
					International migrant	Internal migrant	Ethnicity	Health outcomes	Physio-logical measures	Risk / protective factors	Perception / behaviour	Healthcare access / utilization
1	Al-Bouwarthan, M, 2020 [33]	Saudi Arabia	Construction worker	Cohort	100%	0%	100%	X	X	-	-	-
2	Al-Sayyad, A. S., 2014 [34]	Bahrain	Outdoor worker	Cross-sectional	100%	0%	NA	-	-	-	-	X
3	Albu, I., 2023 [35]	USA	Farmworker	Cohort	At least 86%	NA	Latino: 100%	X	X	-	-	-
4	Arcury, T. A., 2015 [36]	USA	Farmworker	Cross-sectional	100%	100%	Latino: 100%	X	-	X	X	-
5	Arcury, T. A., 2020a [37]	USA	Farmworker	Cross-sectional	18.8%	17.80%	Latino: 100%	X	-	X	X	-
	Arcury, T. A., 2020b [38]			Cross-sectional	NA	17.80%		X	-	X		
	Arcury, T.A., 2019 [39]			Qualitative	50%	46.70%		-	-	-	X	-
	Arnold, T., 2021 [40]			Qualitative	NA	46.70%		X	-	-	X	-
	Arnold, T., 2020 [41]			Mixed methods	NA	Quant: 14% Qual: 46.7%		X	-	-	-	-
	Quandt, S. A., 2019 [42]			Qualitative	50%	46.70%		X	-	-	-	-
6	Bandala, ER, 2022 [43]	USA	Outdoor worker	Cross-sectional	NA	NA	White A. American Latino Asian	X	-	-	-	-
7	Bates, G.P., 2010 [44]	United Arab Emirates	Construction worker	Cross-sectional	100%	0%	NA	-	X	-	-	-
8	Bates, G.P., 2008 [45]	United Arab Emirates	Construction worker	Cross-sectional	100%	0%	NA	-	X	-	X	-
9	Bethel, J.W., 2014 [46]	USA	Farmworker	Cross-sectional	97%	100%	Latino: 99%	-	-	-	X	-
	Bethel, J.W., 2017 [47]				94.9%	100%	Latino: 99%	-	-	-	X	-
10	Boonruksa, P., 2020 [48]	Thailand	Farmworker	Case control	NA	100%	NA	X	X	-	X	-
11	Chavez Santos, E., 2022 [49]	USA	Farmworker	Case control	96%	NA	NA	-	-	X	X	-
12	Cheney, A. M., 2022 [50]	USA	Farmworker	Qualitative	99%	NA	Latino: 94.7%	-	-	-	X	-
13	Courville, M.D., 2016 [51]	USA	Farmworker	Qualitative	96%	NA	Latino: 96%	X	-	-	X	-
14	Culp, K., 2019 [52]	USA	Farmworker	Cross-sectional	100%	NA	Latino: 100%	X	X	X	X	X
15	Dong, X. S., 2019 [53]	USA	Construction worker	Cohort	31.7%	NA	Hispanic NH white NH black Other	X	-	-	-	-
16	Fleischer, N. L., 2013 [54]	USA	Farmworker	Cross-sectional	NA	100%	Latino: 79.5%	X	-	X	X	-
17	Flocks, J., 2013 [55]	USA	Farmworker	Qualitative	100%	NA	Latino: 80%	X	-	-	X	-
18	Gelaye, K. A., 2021a [56]	Ethiopia	Farmworker	Cross-sectional	NA	100%	NA	-	X	-	-	-
	Gelaye, K.A., 2021b [57]							-	X	-	-	-
	Gelaye, K.A., 2020 [58]							-	X	-	-	-
19	Girard, O., 2021 [59]	Qatar	Outdoor worker	Cross-sectional	100%	NA	NA	-	X	-	-	-
20	Grzywacz, J. G., 2019 [60]	USA	Farmworker	Case control	100%	65%	Latino: 100%	-	-	-	X	-
21	Gubernot, D. M., 2015 [61]	USA	Farm and construction worker	Cross-sectional	NA	NA	Non-Hispanic Hispanic	-	-	X	-	-
22	Hofmann, J. N., 2009 [62]	USA	Farmworker	Cross-sectional	68.4%	NA	Hispanic: 91.5%	-	-	-	X	-
23	ILO, 2023 [63]	Lebanon	Farmworker	Qualitative	93%	NA	NA	X	-	-	X	-
24	Ioannou, L. G., 2023 [64]	Cyprus	Farmworker	Case control	74%	NA	NA	-	X	-	-	-
25	Kearney, G. D., 2016 [65]	USA	Farmworker	Cross-sectional	100%	100%	Latino: 100%	X	-	X	X	-
26	Kearney, G.D., 2020 [37]	USA	Outdoor worker	Cross-sectional	100%	NA	Latino: 100%	X	-	-	-	-
27	Lam, M., 2013 [66]	USA	Farmworker	Qualitative	97%	NA	Latino: 100%	-	-	-	X	-
28	Langer, C. E., 2021 [67]	USA	Farmworker	Cross-sectional	91.9%	NA	Latino: 100%	-	X	-	-	-
29	López-Gálvez, N., 2020 [68]	Mexico	Farmworker	Case control	0%	100%	NA	-	X	-	X	-
	López-Gálvez, N., 2021 [69]			Case control				-	X	-	X	-
30	Lundgren-Kownacki, K., 2018 [70]	India	Outdoor Worker	Cross-sectional	NA	100%	NA	X	X	-	X	-
31	Luque, J. S., 2020 [71]	USA	Farmworker	Cross-sectional	98%	NA	Hispanic: 100%	X	-	-	X	-
32	Luque, J. S., 2019 [72]	USA	Farmworker	Qualitative	100%	41%	Hispanic: 100%	X	-	-	X	X
33	Mac, V., 2021 [73]	USA	Farmworker	Cross-sectional	98.20%	NA	NA	-	X	-	-	-
34	Marquez, D., 2023 [74]	USA	Farmworker	Case control	94%	NA	NA	-	-	-	X	-
35	McQueen, S.L., 2012 [75]	USA	Farmworker	Cross-sectional	100%	NA	Latino: 100%	X	X	-	X	-
36	Messeri, A., 2019 [76]	Italy	Outdoor worker	Cross-sectional	36.5%	NA	NA	-	-	-	X	-
37	Mirabelli, M. C., 2010 [77]	USA	Farmworker	Cross-sectional	NA	100%	Latino: 100%	X	-	-	X	-
38	Mitchell, D. C., 2017 [78]	USA	Farmworker	Cross-sectional	91.80% NA	NA	Latino: 97.9%	-	X	-	-	-
	Mitchell, D. C., 2018 [79]		Farmworker	Cross-sectional		NA		-	X	-	-	-
39	Mix, J., 2018 [80]	USA	Farmworker	Cross-sectional	98.00%	NA	NA	-	X	-	X	-
40	Mizelle, E., 2022a [81]	USA	Farmworker	Qualitative	100.00%	H-2A visa*: 100%	Latino: 100%	-	-	-	X	-
	Mizelle, E., 2022b [82]		Farmworker	Mixed methods				-	X	-	X	-
41	Moohialdin, A.S.M., 2022 [83]	Saudi Arabia	Construction worker	Cross-sectional	100%%	NA	NA	-	X	-	-	-
42	Morera, M.C., 2020 [84]	USA	Farmworker	Qualitative	At least 75%	H-2A visa*: 75%	Latino: 100%	X	-	-	X	X
43	Moyce, S., 2016 [85]	USA	Farmworker	Cross-sectional	94.60%	NA	NA	X	X	-	-	-
	Moyce, S., 2017 [86]				94.70%			X	X	-	-	-
	Moyce, S., 2019 [87]				92.57%			X	X	-	-	-
	Moyce, S., 2020 [88]				92.40%			X	X	-	X	-
44	Mutic, A. D., 2018 [89]	USA	Farmworker	Cross-sectional	98%	NA	Latino: 98%	X	-	X	-	-
45	Odame, E.A., 2019 [90]	USA	Farmworker	Cross-sectional	91%	100%	NA	X	-	X	-	-
46	Petitti, D. B., 2013 [91]	USA	Outdoor worker	Case control	NA	NA	NH white Hispanic N. American A. American Others	X	-	-	-	-
47	Pradhan, B., 2019 [92]	Qatar	Construction worker	Cross-sectional	100%	0%	NA	X	-	-	-	-
48	Quiller, G., 2017 [93]	USA	Farmworker	Cross-sectional	96%	NA	Latino: 98%	X	X	-	-	-
	Spector, J., 2018 [94]	USA	Farmworker	Cross-sectional				-	X	-	-	-
49	Riccò, M., 2019a [95]	Italy	Outdoor worker	Cross-sectional	8.4%	NA	NA	X	-	X	-	-
50	Riccò, M., 2019b [96]	Italy	Outdoor worker	Cross-sectional	4.2%	NA	NA	X	-	-	-	-
51	Smith, D. J., 2021 [97]	USA	Farmworker	Cross-sectional	92%	100%	Latino: 100%	X	-	-	X	-
52	Spector, J., 2015 [98]	USA	Farmworker	Cross-sectional	93%	NA	Latino: 99%	X	-	X	X	-
53	Stoecklin-Marois, M., 2013 [99]	USA	Farmworker	Cross-sectional	98%	NA	Latino: 100%	-	-	-	X	-
54	Stone, W.A., 2022 [100]	USA	Outdoor worker	Qualitative	NA	100%	NA	-	-	-	X	-
55	Taylor, E. V., 2018 [101]	USA	Outdoor worker	Cross-sectional	NA	NA	Hispanic non-Hispanic	X	-	-	-	-
55 56	Vega-Arroyo, A.J., 2018 [102]	USA	Farmworker	Cross-sectional	90%	NA	Latino: 96.9%	-	X	-	X	-
	Vega‐Arroyo, A.J., 2019 [103]							-	X	-	-	-
57	Venugopal, V., 2016 [104]	India	Construction worker	Mixed methods	NA	100%	NA	X	X	-	X	-
58	Villanueva-Gómez, R., 2023 [105]	Canada	Farmworker	Mixed methods	100%	0%	Latino: 100%	X	-	-	-	-
59	Wagoner, R. S., 2020 [106]	Mexico	Farmworker	Cross-sectional	0%	100%	NA	-	X	-	-	-
60	Zhang, K., 2016 [107]	USA	Farmworker	Cross-sectional	NA	39.40%	Latino: 88.7%	-	-	-	-	X

The “#” section includes the number of unique studies, while the “reference paper” section includes all the papers using (partially) unique data from these studies. | † In line with the inclusion criteria, percentages from 85% are considered a representative sample for the corresponding MEM category. When the percentage is lower this means that the paper presents separate data for MEM and non-MEM categories. | * The H-2A visa program is a program that allows agricultural employers to bring foreign nationals to the United States to fill temporary agricultural jobs [108]. | ILO: International Labour Organization; MEM: migrant and ethnic minorities; NH: non-Hispanic; N. American: native American; A. American: African American.

### Heat Exposure

**Fig. 3:**
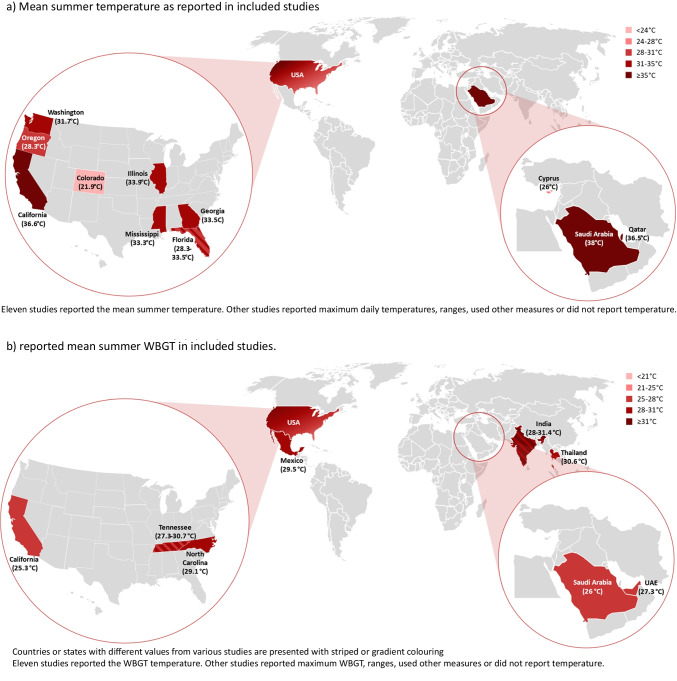
Reported temperatures in included studies. **a**) Mean summer temperature as reported in included studies. Eleven studies reported the mean summer temperature. Other studies reported maximum daily temperatures, ranges, used other measures or did not report temperature. **b**) reported mean summer WBGT in included studies. Countries or states with different values from various studies are presented with striped or gradient colouring. Eleven studies reported the WBGT temperature. Other studies reported maximum WBGT, ranges, used other measures or did not report temperature

Thirty-seven studies included definitions of heat exposure, the majority using publicly available data from nearby weather stations during the period of data collection. The measures that were mostly reported were wet-bulb globe temperature (WBGT) and mean daily temperature (both *n* = 11). Reported mean daily temperatures ranged from 21.9 °C (Colorado) to 38 °C (Saudi Arabia), while mean WBGT varied from 25.3 °C (California) to 31.4 °C (India) (Fig. 3).

### Heat-Related Health Outcomes

#### Physiological Measures

Twenty studies included data on vital signs, including body temperature (*n* = 12), heart rate (*n* = 9), metabolic work rate (*n* = 8), body mass (*n* = 7) and blood pressure (*n* = 5). Twelve studies included biological measures of dehydration and kidney function using urine specific gravity (*n* = 8), estimated Glomerular Filtration Rate (eGFR) (*n* = 4) or Creatinine (*n* = 4).

Table 2 provides an overview of studies assessing heat stress, dehydration and kidney function based on physiological measures. Heat stress prevalence varied across studies and type of physiological measure (body temperature: 0–67%; heart rate: 11–33%). Dehydration based on body mass was found among 11–33% of workers, while Urine Specific Gravity (USG) measures indicating clinical dehydration (USG > 1.020) varied widely (0–100%) and indicating severe dehydration (USG > 1.030) ranged from 13–53%. Among studies assessing kidney function, prevalence of kidney damage and kidney disease were 5–8% and 1% respectively based on Estimated Glomerular Filtration Rate (eGFR) and 15–33% of workers had Acute Kidney Injury (AKI).

**Table 2:** Heat stress, dehydration and kidney injury based on physiological measures

Topic	Measure	Criterium	Percentage of workers meeting criterium
Heat strain	Body temperature	Body temperature > 38 °C	0% [75], 1% [106], 45% [103], 67%[73]
		Body temperature > 38.5 °C	7% [67], 8.3% [78] 16% [73]
		> 1 °C increase over work shift	38.2% [88]
	Heart rate	Heart rate (HR) outside of acceptable HR ranges (HR_min_- HR_max_)*	11% [83]
		Sustained heart rate [180 – (age in years)] bpm for five minutes or more	18% [78]
		Heart rate > 110 bpm after 1 min of rest	33% [75]
	Body temperature and heart rate	Sustained heart rate [180 – (age in years)] bpm for several minutes or body temperature > 38.5 °C	28.3% [94]
Dehydration (after work shift and / or season)	Body mass	Losing more than 1.5% of body weight	10.6% [88], 11.8% [78], 14.3% [103], 33% [75]
	Urine Specific Gravity (USG)	USG > 1.020 (clinical dehydration)	0% [45], 81% [80], 83% [69], 87.8% [48], 100% [82]
		USG = / > 1.026	14–31% [44]
		USG > 1.030 (severely dehydrated)	13% [80], 53.3% [48],
Kidney damage	Estimated Glomerular Filtration Rate (eGFR)	eGFR < 90 mL/min/1.73m2	4.5% [85], 7.9% [69]
Kidney disease	Estimated Glomerular Filtration Rate (eGFR)	eGFR < 60 mL/min/1.73m2	1% [69]
Acute Kidney Injury (AKI)	Serum creatinine	Serum creatinine ≥ 0.3 mg/dl or at least 1.5 increase	14.8% [85], 15% [35], 33% [80]

*HR_min_ = HR_min rest_ – (2* HR_SD rest_)_,_ HR_max_ = HR_max rest_ – (2* HR_SD rest_) | AKI: Acute Kidney Injury; Bpm: Beats Per Minute; eGFR: Estimated Glomerular Filtration Rate; HR: heart rate; USG: Urine specific gravity

Several studies (*n* = 16) assessed changes in physiological measures over working shifts or seasons (Table 3). Most studies found that body temperature (*n* = 2) and blood pressure (*n* = 2) increased over work shifts or over the working season, while findings for heart rate were contrasting with three studies showing an increase and three others not detecting any change. All studies measuring body weight found a decrease over the work shift, suggesting reduced hydration levels. Regarding kidney function, eGFR reduced over the work shift and season, while serum creatinine increased, both indicating reduced kidney function. Heterogeneous results were found for metabolic work rate, the rate at which the body uses energy to perform physical tasks, over seasons. One study found that the metabolic work rate increased with the temperature [68], another showing less physical activity in the hot season [59] and a third study showing a reduced metabolic rate during hotter seasons, while also reaching moderate and heavy rates [106]ç.

**Table 3:** Number of studies reporting short- and long-term changes in physiological measures

Measures	Over shift (short-term)			Over season (long-term)		
	Increased	No difference	Decreased	Increased	No difference	Decreased
Blood pressure	1 [48]		1 [59]	2 [48, 68]		1 [59]
Heart rate	2 [48, 59]	1 [45]		1 [68]	2 [59, 70]	
Body temperature	3 [48, 75, 78]			2 [59, 68]	1 [106]	
Body mass			4 [75, 78, 88, 103]			
Urine Specific Gravity	3 [48, 80, 82]	1 [94]		1 [69]	1 [106]	
Estimated Glomerular Filtration Rate (eGFR)			1 [80]			1 [68]
Creatinine	2 [44, 80]			2 [33, 69]		

Two studies compared physiological measures between migrants and non-migrant farmworkers. A Californian study found no difference in body temperature between international migrants and non-international migrants [67]. Among farmworkers in Cyprus, time spent with a body temperature above 38 °C was higher for migrants from low and middle income countries (LMIC) (27.7%) and upper middle income countries (UMIC) (17%), compared to native workers (13.4%). Workers from LMIC also worked at a higher average intensity (LMIC: 198.0 W/m^2^, UMIC: 195.6 W/m^2^, HIC: 182.0 W/m^2^) and wore more clothing [64].

#### Heat-Related Illnesses

Twenty-two quantitative and eight qualitative studies evaluated prevalence of or experience with HRI symptoms among outdoor workers. All qualitative and 17 quantitative studies were conducted in the US and Canada among Latino migrant farmworkers (Table 4). In most quantitative studies, the majority of participants were male, accounting for 23–100% of the study samples across studies. Questions about HRI symptoms varied in the time window considered, with the majority asking about the last week (*n* = 8), while others focused on the day of the survey (*n* = 3) or on the long term (current season, past three months or ever). On average, among Latino migrant farmworkers in the US, 48.8% had experienced at least one and 27.7% at least three HRI symptoms. The most common symptoms were headache (41.3%), heavy sweating (33.9%) and muscle cramps (27.7%), followed by dry skin or skin rash (20.6%) and extreme weakness or fatigue (16.1%) (mean weighted prevalence). In addition, participants in qualitative studies mentioned experiencing “feverish chills” and kidney pain and pregnant women reported “*feeling dizzy and wanting to throw up because you are bent over while you are pregnant*” [55, 72, 81]. Moreover, several workers mentioned having to stop working because they felt unwell [40, 41, 72, 84].

**Table 4:** Prevalence of heat-related illness symptoms among Latino migrant farmworkers in the US and Canada

				Number of symptoms		Type of symptoms								
Reference	Migrant type	Sample size	Asked about HRI	At least 1	At least 3	Headache	Heavy sweating	Muscle cramps	Dry skin / skin rash	Dizziness	Extreme weakness or fatigue	Nausea / vomiting	Confusion	Fainting
Albu, L, 2023 [35]	International	113	During that workday	36.3%	-	-	-	-	-	-	-	-	-	-
Arcury, 2015 [36]	Internal	101	In previous 3 months	35.6%	-	-	-	16.8%	21.8%	10.9%	-	6.9%	8.9%	-
Arcury, 2020a & 2020b* [37, 38]	NA	202	In past year	45.5%	-	-	-	21.8%	17.3%	25.7%	-	7.4%	5.0%	1.5%
Bethel, 2014 [46]	International and internal	100	In past week	64.0%	11.0%	24.0%	50.0%	9.0%	10.0%	7.0%	14.0%	2.0%	3.0%	1.0%
Culp, 2019 [52]	International	148	During that workday	-	-	-	-	7.4%	3.1%	5.4%	-	4.0%	6.0%	-
Fleischer, 2013 [54]	Internal	402	In past week	60.6%	33.8%	50.8%	-	33.7%	44.9%	24.6%	-	16.7%	15.5%	4.4%
Kearney, 2016 [65]	Internal	158	In past week	72.3%	-	43.6%	37.6%	35.7%	12.1%	13.5%	17.9%	8.5%	1.4%	4.3%
Kearney, 2020 [37]	International	57	In past year	-	-	33.9%		7.0%	5.3%	-	-	-	-	1.0%
Luque, 2020 [71]	International	101	In past week	18.0%	5.0%	14.0%	12.0%	1.0%	6.0%	6.0%	3.0%	3.0%	-	-
McQueen, 2012 [75]	International	85	In past week	-	-	48.2%	-	30.6%	-	30.6%	-	16.5%	-	-
Mirabelli, 2010 [77]	Internal	281	Ever experienced	40.0%	-	-	-	-	-	-	-	-	-	-
Mutic, 2018 [89]	International	198	In past week	83.0%	40.0%	58.0%	66.0%	30.0%	-	32.0%	-	24.0%	9.0%	10.0%
Odame, 2019 [90]	International and internal	292	NA	18.8%	-	-	-	-	-	-	-	-	-	-
Quiller, 2017[93]	International	46	In past week	52.2%	-	-	-	-	-	-	-	-	-	-
Smith, 2021 [97]	International and internal	60	During that workday	68.0%	12.0%	22.0%	50.0%	25.0%	-	10.0%	-	3.0%	0.0%	0.0%
Spector, 2015 [98]	International	97	In past week	45.4%	-	19.6%	28.9%	1.0%	3.1%	3.1%	2.1%	2.1%	-	0.0%
Villanueva-Gómez, 2023 [105]	International	9	In the current season	66.7%	-	55.5%	44.4%	-	-	-	-	-	-	-
**# Studies including symptom**				14	5	10	7	12	9	11	4	11	8	8
**Weighted average (95% CI)**^§^				**48,0.8%** (47.1, 50.5)	**27.7%** (26.7, 28.7)	**41.3%** (40.0, 42.6)	**33.9%** (32.9–34.9)	**22.1%** (20.8, 23.4)	**20.9%** (19.8, 22.0)	**18.4%** (17.2, 19.6)	**16.1%** (15.6, 16.6)	**10.8%** (9.8, 11.8)	**8.4%** (7.6, 9.2)	**3.4%** (2.9, 3.9)

* Study participants were minors | ^§^ 95% Confidence Intervals calculated based on binomial distribution [31] | HRI: heat-related illnesses

One mixed-methods study among farmworkers in the US included minors [37, 38, 40–42]. In addition to the abovementioned symptoms, minors mentioned nosebleeds and getting frustrated because of the heat [40, 41]. Some minors also mentioned having seen other people faint and being frightened because it looked like they were dead [40–42]. This study among minors compared internal migrants with non-migrants and suggested that non-migrants had a lower chance of HRI symptoms (OR 0.57, 95% CI 0.20, 1.63), although not statistically significant [38].

In addition to the US studies, four studies reported HRI symptoms in internal migrants in Thailand [48], Ethiopia [57] and India (*n* = 2) [70, 104]; four studies reported HRI among international migrants in Saudi Arabia (*n* = 2) [33, 83], Italy [95] and Lebanon [63] and one study assessed heat-related deaths in international migrants in Qatar [92]. The studies on internal migrants found high prevalence of HRI symptoms, including 57–91% experiencing (extreme) weakness [48, 57, 70], 43–100% heavy sweating [48, 57, 70, 104], 40–67% headache [48, 57, 70, 104], 40–45% dizziness [48, 57], 24–50% dry skin or skin rash [57, 70, 104], 21–52% muscle cramps [48, 57, 70], and 8–15% reporting having fainted [48, 57, 70]. Finally, in Ethiopia 60.9% reported having experienced at least three symptoms in the past week [57]. A study among Indian migrants in Saudi Arabia reported changes in symptoms over summer. Even though temperatures were similar at both times, muscle cramps were reported more frequently at the beginning of summer (28% beginning; 0% end) while headache (23% beginning; 54% end) and fever (15% beginning; 37% end) was reported more at the end of summer [33]. In Saudi Arabia, no one reported occupational heatstroke while 63% of Syrian farmworkers in Lebanon reported sun or heat stroke [63, 83]. A study in Italy included a small sample of international migrant workers (*n* = 11) where 18% reported at least three HRI symptoms [95]. A study on mortality among Nepali workers in Qatar showed that in the hot season (WBGTmax 30 °C) 58% of deaths were caused by cardiovascular disease, while in the cold season (WBGTmax 20 °C) this was only 22%. For each 1 °C increase of WBGT the mortality rate went up by 5.5/100,000 among Nepali workers [92].

#### Heat-Related Injuries and Deaths

Two studies compared occupational injuries while working in a hot environment between MEM and non-MEM. One US study calculated nonfatal occupational heat-related injuries and illnesses among outdoor workers per 100,000 inhabitants as a function of ethnic groups. Most injuries occurred among whites, followed by Latinos in Arizona (3,990 whites; 3,460 Latinos) and Nevada (2,320 whites; 1,700 Latinos) while for California this was the opposite (41,440 Latinos; 21,450 whites), which is likely a representation of the outdoor working populations in these states [43]. In Northern Italy, during the Ramadan period migrant workers from the Eastern-Mediterranean Region were at higher risk (OR: 1.42, 95%CI: 1.10–1.84) for occupational injuries during heat waves (at least three consecutive days with a maximum temperature ≥ 35 °C), compared to Western European workers. However, they had a lower risk (OR: 0.74, 95%CI: 0.59–0.92) on summer days (maximum temperature > 25 °C) and no significant differences were found during regular days or summer days with tropical nights (maximum temperature > 25 °C and minimum temperature > 20 °C) [96].

In the US, the risk of heat-related death was increased in Hispanics, versus non-Hispanics when working in agriculture (OR: 3.4, 95%CI 2.0–5.8) or construction (OR: 1.7, 95%CI: 1.1–2.6) [61]. Likewise, compared to all construction workers in the US, Hispanics had a 1.21 higher risks of heat-related death, especially when born in Mexico (1.91 times higher) [53]. In Arizona, US, compared to other professions, Hispanics did not have a significantly higher risk of dying from heat-related deaths when working in construction (OR: 1.20, 95%CI: 0.46–3.11) or agriculture (OR: 2.04, 95%CI: 0.80–5.20) [91]. In the US, non-US citizens more often died after heat exposure at a farm compared to US citizens (19.8% of all heat-related deaths among non-US citizens versus 1.5% among US citizens), while an opposite trend was observed for deaths after heat exposure at an industrial or construction site (0.5% for non-US citizens versus 1.6% for US citizens) [101].

### Risk and Protective Factors for Heat-Related Health Outcomes

Table 5 shows the results of 10 studies that reported on risk and protective factors for HRI among MEM outdoor workers. The effects of gender and age were inconclusive, one study reporting female gender as a risk factor[89] while three other studies did not find a significant effect [49, 90, 98]. Likewise, one study found older age to be a risk factor [49], while another study found it to be a protective factor [98] and three other studies did not find a significant correlation [52, 89, 90]. The majority of factors were only investigated in one single study, with higher maximum heat index at work [49, 90] and having to walk more than three minutes to a toilet [49, 98] being the only significant risk factors included in two studies. Being an internal or international migrant was not found to be associated with HRI, however, both studies reporting this had very small migrant samples [37, 95].

**Table 5:** Number of studies reporting on risk and protective factors^§^ for heat-related illness among MEM outdoor workers

Factor	Risk	Protective	No effect
Socio-demographic	**-**	**-**	**-**
Female gender	1 [89]	-	3 [49, 90, 98]
Older age	2* [38, 49]	1 [98]	3 [52, 89, 90]
Education level	-	-	2 [54, 89]
Migration-related factors	-	-	-
Nationality	-	-	1 [89]
Being internal migrant	-	-	1* [37, 41]
Being international migrant	-	-	1 [95]
Time staying in region this year	-	-	1 [54]
Health-related factors (self-reported)	-	-	-
Alcohol consumption	-	-	2 [54, 89]
Smoking	-	-	1 [89]
BMI	-	-	2 [49, 89]
General health	-	-	1 [98]
Hypertension or diabetes	-	-	2 [89, 98]
Previous HRI	-	-	1 [49]
Employment-related factors	-	-	-
Working outdoors	-	-	1 [90]
Piece rate payment type	1 [98]	-	-
Company size	-	-	1 [49]
Not having a H-2A visa†	1 [49]	-	-
10 + years of working experience	1 [49]	-	1 [90]
Number of working days a week	-	-	2 [54, 89]
Number of working hours a day	-	-	2 [54, 89]
Work type (nursery, fernery or crop)	-	-	1 [89]
Environmental factors	-	-	-
Higher max heat Index at work	2 [49, 90]	-	-
Maximum physiological strain index (PSI)	-	-	1 [49]
Tasks	-	-	-
Topping tobacco	1 [36]	-	-
Barning or leading tobacco	1 [36]	-	-
Working in harvest	1 [36]	-	-
Loading / packing outside	-	-	1 [54]
Working at medium / high intensity level	1 [49]	-	-
Facilities at work	-	-	-
Previous HRI training	-	-	2 [49, 90]
Access to shade	-	-	1 [54]
> 3-min work to the toilet	2 [49, 98]	-	-
Access to regular breaks	-	1 [54]	-
Access to medical attention	-	1 [54]	-
Preventive behaviour	-	-	-
Change working duties in hot weather	-	-	1 [54]
Limiting time in the sun	-	1 [65]	-
Take breaks in shaded areas	-	1 [54]	-
Taking extra breaks	-	-	1 [98]
Going somewhere to cool down	-	-	1 [54]
Drinking more water	-	-	1 [54]
Drinking more juice	-	-	1 [54]
Drinking more sports drinks	1 [54]	-	-
Drinking more soda	1 [54]	-	-
Drinking caffeine	-	-	1 [98]
Spending time after work in extremely hot house	1 [36]	-	-
Having air conditioning at home	1 [49]	-	-
Clothing	-	-	-
Wearing wet clothes	1 [36]	-	-
Wearing wet shoes	1 [36]	-	-
Not wearing a hat	1 [65]	-	-
Wearing a shirt with collar	-	-	1 [65]
Wearing protection over face	-	-	1 [65]
Wearing long pants	-	-	1 [65]
Wearing sunglasses	-	-	1 [65]
Wearing sunscreen	-	-	2 [54, 65]
Wearing long-sleeved shirts	-	-	1 [65]

BMI: Body mass index; HR: Heat-related illnesses; MEM: migrant and ethnic minorities | * One of the studies conducted among minors | † The H-2A visa program is a program that allows agricultural employers to bring foreign nationals to the United States to fill temporary agricultural jobs [108]. | ^§^ The direction of the association was based on the comparison of symptom prevalence across different strata of the factor or regression coefficients (from linear or logistic models) and statistical significance based on p-value threshold of 0.05 or 95% Confidence Interval (CI) entirely excluding 0 (for linear models) or 1 (for logistic models).

### Knowledge, Perception and Behaviour Related to Occupational Heat Stress

Studies including findings on workplace facilities and behaviours to prevent heat-related problems used heterogeneous measures, making comparison difficult. Data from studies among MEM farmworkers in the US that used comparable measures are presented in Table 6. When available, data from other studies are described in the text. An extensive synthesis from the qualitative results is available in supplementary Table 2.

**Table 6:** Frequencies of heat-related knowledge and perception, HRI training, protective behaviours, and workplace characteristics among US-farmworkers

				Training	Knowledge and perception		Hydration		Breaks			Activities		
Reference	Migrant type	Latino (> 85%)	Sample size	Received HRI training	Concerned about HRI / heat	High HRI knowledge score (vs low)	Drink more water when hot	Employer provides fluids	Take breaks in shade when hot	Go to cool place when not working	Comfortable taking break to drink water	Change work hours	Change work activities	Gradually increased # working hours start season
Arcury, 2015	Internal	Yes	101	-	-	-	87%	-	88,2%	29,4%	-	25%	20,6%	-
Bethel, 2017	International and internal	Yes	197	44%	67%	79%	-	89%	-	-	78%	-	-	41,1%
Chavez Santos, 2022	International	NA	75	72%	-	-	-	-	–	-	-	-	-	-
Culp, 2019	International and internal	Yes	148	24%	-	-	-	-	-	-	-	-	-	-
Fleischer, 2013	Internal	No	405	-	-	-	96%	97%	79,8%	43,8%	-	37%	36,2%	-
Grzywacz, 2019	International and internal	Yes	127	21%	-	-	-	-	-	-	-	-	-	-
Hofmann, 2009	NA	Yes	389	-	72,5%	-	-	-	-	-	-	-	-	-
Kearney, 2016	Internal	Yes	158	-	58,3%	-	-	-	-	-	96%	-	-	-
Luque, 2020	International	Yes	101	-	41%	43%	67%	-	62%	-	-	21%	23,0%	58%
Marquez, 2023	International	NA	83	69%	-	-	-	-	-	-	-	-	-	-
McQueen, 2012	International and internal	Yes	85	-	84%	-	95%	-	48%	13%	-	-	-	-
Mirabelli, 2010	Internal	Yes	281	-	-	-	98%	-	81%	2%	-	37%	34,0%	-
Mix, 2018	International	Yes	192	-	-	-	-	90%	-	-	-	-	-	-
Spector, 2015	International	Yes	97	33%	71%	-	83%	-	-	-	74%	-	-	34%
Stoecklin-Marois, 2013	International	Yes	474	92%	53%	70%	-	88%	-	-	98%	-	-	-
Vega-Arroyo, 2018	International	Yes	288	-	54,2%	-	-	-	-	-	-	-	-	-
***#* Studies including factor**				7	8	3	6	4	5	4	4	4	4	3
**Weighted average (95% CI)**^§^				**60.4%** (59.7, 61.1)	**60.9%** (59.7, 62.1)	**67.3%** (66.5, 68.1)	**91.7%** (91.2, 92.2)	**91.1%** (91.1, 92.3)	**85.2%** (84.4, 86.0)	**25.7%** (25.0, 26.4)	**92.0%** (91.5, 92.5)	**34.0%** (33.1, 34.9)	**32.2%** (31.4, 33.0)	**43.7%** (43.1, 44.3)

#### Heat Related Knowledge and Perception

Risk perception was assessed both quantitatively and qualitatively. Across US-studies, 60.9% (range: 41–84%) of MEM farmworkers reported being concerned about heat (Table 6) and in qualitative studies MEM workers expressed concerns as well. Among farmworkers in Washington state, Latinos reported to be more concerned about working in hot weather than non-Latinos (OR: 1.90, 95%CI: 0.44–8.25), but this difference was not significant [62]. In Italy, international migrant workers perceived the workplace temperature as less hot compared to native workers [76].

In US studies, 60.4% of MEM farmworkers received HRI training at their workplace [47, 49, 52, 60, 74, 98, 99]. Three studies evaluated HRI knowledge with workers having high knowledge score ranging from 43 to 79% (Table 6) [46, 71, 99]. Other countries show worse realities, with only 5% of Syrian workers in Lebanon reported having had occupational safety and health training [63]. In Italy, compared to natives, migrants reported having received less information on how to act during heat waves but said to be more satisfied about the workplace measures to protect them from heat exposure [76]. In Saudi Arabia, workers said they never received a warning when temperatures or humidity would reach high levels [83].

Qualitative studies showed that in the US, MEM farmworkers could mention causes and symptoms of HRI, although some incorrect symptoms were mentioned as well. Some were aware of prevention strategies although others considered HRI unavoidable [84]. Workers reported knowing what to do in.

case of HRI, but there was disagreement for specific measures such as cooling with ice [66]. Some workers had heard of RHI but were not aware of HRI symptoms and prevention strategies beyond resting and drinking water and some expressed lacking information about HRI [50, 51, 81].

Grey cells: factors related to workplace characteristics; White cells: worker perception and behaviours. ^§^ 95% Confidence Intervals calculated based on binomial distribution [31]

#### Fluid Intake

In the US, on average 90.1% of MEM farmworkers reported that employers provided water [47, 54, 80, 99] and 91.7% of workers reported drinking more water when it was hot (Table 6) [36, 54, 71, 75, 77, 98]. In Lebanon, 80% reported that water was available at the work place [63]. In the US, the majority of farmworkers reported drinking water during the work shift (95%−98.5) [47, 54, 80, 98], drinking every 15 min (96.7%) [82] or at least once per hour (78–83%) [47, 98], with an average of 10.7 times a day [99]. US farmworkers reported consuming 2.1–2.8 L of water per day [82, 88, 97], UAE construction workers 5.4 L, [45] while the majority (88.9%) of farmworkers in Mexico consumed less than 4 L [68] and most Thai farmworkers consumed 1.1–3 L (44.4%) or 3.1–5 L (31.1%) per day [48]. Other drinks consumed at work among farmworkers in the US were mainly sports drinks (4.7–69%) and sodas (19.6–83.8%) [47, 52, 54, 80, 98]. Among farmworkers in Mexico, 77.3% had recently consumed sweetened drinks [54].

While construction workers in India did not have access to a toilet at their workplace [104], the majority of farmworkers in Lebanon (70%) [63] and the US (95.8–98%) did [54, 80]. However, 35.6–84% of MEM workers in US had to walk for more than 3 min to reach the toilet [47, 82, 98]. Despite acknowledging the importance of drinking water [81], MEM-workers provided several reasons to limit water intake, including low perceived water quality of water at the work site and not wanting to interrupt work to go to the toilet [55, 66, 81, 100]. To cool down workers mentioned to wet their heads but said drinking very cold water could be dangerous [51, 72].

#### Working Hours and Breaks

Regarding heat acclimatization at the start of the season, on average 43.7% of MEM farmworkers in the US reported gradually increasing their working hours at the beginning of the summer [47, 71, 98]. During hot days, 34% changed working hours and 32.3% changed working activities (Table 6) [36, 54, 71, 77]. In qualitative studies, workers mentioned starting earlier or getting longer lunch breaks to avoid working during the hottest hours [81, 105].

Five studies reported on resting schemes during the working day. In India, construction workers did not have a structured resting scheme [104], while in the US, the majority reported having access to lunch breaks (93.3–99%) and/or regular breaks (66.1–89.7%) [47, 98, 99]. Adult workers in North Carolina reported having a 30 min lunch break and two additional 5–15 min breaks on a 8–12 h workday, while minors mentioned having two breaks on a nine-hour workday [42, 81]. In the US, almost all (92%) participants reported feeling comfortable taking drinking breaks [46, 65, 98, 99]. Most farmworkers (85.2%) reported taking breaks in the shade when it was hot [36, 54, 71, 75, 77] and 25.7% went to a cool place when not working (Table 6) [36, 54, 75, 77]. Brick kiln workers in India reported that 75% took breaks in the shade when hot [70]. In the US, 72.8–96% of farmworkers reported having access to shaded areas [47, 54, 98, 99], mainly from trees (69%−92%) [47, 65, 71]. Sometimes shade structures (13–22%) [47, 65, 71, 98] or rest stations (10–16%) [47, 65, 71, 98] were available, while air-conditioned spaces were rarely provided (1.5–2%) [47, 71].

Both adults [84] and minors [41, 42] recognized the importance of resting, especially when it was hot, but barriers to taking breaks or slowing down the working pace were mentioned, including supervisors threatening with withholding breaks or salaries when not working fast [41, 42] or being able to earn more when working faster when being paid by the piece [51, 72]. Experiences with supervisors varied but trust appeared to be an essential element for heat safety both in minor and adult workers.

#### Clothing

The majority of MEM farmworkers in the US reported wearing loose or light weighted (81.2–96%) [54, 102], light-coloured (45.7–92%) [47, 71, 98, 102], clothing with long-sleeves (80.5–90%) [47, 54, 65, 71, 98], and long pants (83–98%) [47, 54, 65, 71].

To protect their heads most workers wore baseball caps (76–85.3%) [47, 71, 98, 102], and only a minority wore wide-brimmed hats (9.1–32.6%) [47, 54, 65, 71, 98, 102].

Qualitative studies explained how workers sometimes covered themselves up to prevent the effects from chemicals, despite being aware that it could increase the risk of HRI [66].

### Healthcare Utilization and Access to Healthcare For Health-Related Illnesses and Injuries

Only three quantitative and two qualitative studies provided data on access to healthcare services. In Iowa, US, 2.7% of farmworkers, all Latino and international migrants, reported having gone to the on-site medical clinic for dehydration and heat-related symptoms none of which required hospitalization and/or IV fluid administration [52]. Medical records from a healthcare centre in Colorado, US, reported a daily average of 2.8 visits by internal migrant farmworkers in the summer. Heat was significantly associated to higher risk of clinic visits for all workers (%change = + 88% by comparing 90th to 50th percentile) and for male workers (%change = 118% increase) on hotter days [107]. Finally, a healthcare centre for international migrant workers in Bahrain reported that during a two-week period in summer, 4.8% of visits were related to HRI, of which 95.5% worked fully or partially outdoors [34].

Some barriers to access to healthcare services were identified. In North Carolina, US, MEM workers reported concerns about costs of healthcare. Some mentioned going to free clinics while others reported resting rather than going to the clinic, looking up the symptoms and the treatment on the internet or only said going when very sick [72]. In Florida, workers mentioned that the heat was “normal and part of the job” and therefore they would not usually get treatment for heat-related injuries [84].

## Discussion

This study reviewed the available literature on occupational heat exposure among MEM outdoor workers. This review has the value to integrate both quantitative and qualitative evidence about heat-related risks and prevention measures among MEM workers. Workers’ narratives allowed to interpretate quantitative results mostly from cross-sectional studies. Significant variation in study designs, environmental conditions, study populations and included outcomes and measures impair the possibility to provide a quantitative synthesis, however some main themes emerged.

The weighted prevalence across US studies showed that around half of the MEM workers were suffering from HRI (48.8% experienced at least one HRI symptom and 27.7% at least three), with higher levels in non-US countries. Moreover, HRI among MEM outdoor workers might be underrepresented in the literature as this population can be reluctant to report occupational injuries and illnesses obtained due to their dependence on their employers for work, especially in the case of undocumented workers [109, 110]. No studies found a difference in HRI prevalence between MEM and non-MEM workers but further studies are needed to better address underlying vulnerability factors, including socioeconomic factors, health status, access to healthcare and occupational health and safety factors of MEM compared to non-MEM workers.

Barriers to engaging in HRI preventive behaviours were identified that might be related to MEM status, including fear of supervisors, being paid per unit of crop harvested (working at piece rate) and limited levels of heat training. In addition, perception of heat risk was limited with over a third of MEM reporting not being concerned about HRI or heat in US farmworkers studies. Fear of losing work and therefore accepting working conditions that can be dangerous for one’s health has been reported in multiple studies on migrant farmworkers in the Europe, the US and Canada [23, 111, 112]. Moreover, some MEM farmworkers are paid at a piece rate, which encourages workers to work faster to increase their salary, regardless of heat strain they might be experiencing [113]. A potential explanation for the limited risk-perception in this population could be the relatively low levels of HRI training in the workplace identified in this review. A study from the Gulf region found that lack of safety training was the main reason for occupational accidents among construction workers [114]. However, even when receiving safety and health training, a US study showed that these trainings are often not well adjusted to the educational level, language and culture of migrant farmworkers [115]. For example, in North Carolina, around a quarter of migrant farmworkers who received pesticide training reported not understanding the training [116]. This could explain why having had HRI training was not found to impact HRI levels among MEM outdoor workers in two studies included in the review [49, 90].

There is a lack of studies on utilization of and access to healthcare services for MEM outdoor workers. Although barriers to healthcare access emerged from the qualitative studies, there were no studies identified to quantify these findings. Non heat-related studies among MEM outdoor workers about access to health care suggested limited healthcare utilization and access for this population. In the US, the National Agricultural Workers Survey (NAWS) 2021–2022 results showed that only 24% of foreign-born migrant crop workers and 38% of internal migrant workers had health insurance [117], which could prevent migrant workers from using healthcare services due to concerns about costs, as was mentioned in the findings of this review. Notably, in 2021–2022 only 43% of uninsured crop workers reported having used healthcare services in the previous year compared to 77% of insured workers [117]. A study on healthcare utilization, including workers (indoor and outdoor combined) with occupational HRI in the Southeast region of the US found that, compared to non-Hispanics, Hispanic workers had lower rates of emergency department visits (RR = 0.54, 95% CI 0.5,0.6), while having higher rates of inpatient hospitalizations (RR = 1.5, 95% CI 1.2,1.8) (no information on health insurance status reported) [118]. These findings could indicate that in the US both migrants, especially when undocumented, and Latino workers might face barriers to healthcare services and therefore only go with very severe complaints (resulting in hospitalizations). In the Gulf states, a study among Nepali migrant workers reported 93.5% high levels of health insurance [119]. However, despite having health insurance Bangladeshi migrant workers in the UAE reported barriers including high out of pocket costs as a result of limited insurance coverage and the inability to seek medical care due to wage cuts for lost hours [120]. In Qatar, healthcare utilization of outpatient services by migrant workers was found to be almost twice as low as that of Qataris and an ever bigger difference was found for inpatient services [121].

A limitation in this review was the lack of comparability across studies due to the heterogeneous study populations, definitions and levels of heat exposure, and study outcomes. Many studies did not define the term migrant and in multiple cases it was not clear whether the study population included international migrants, internal migrants or both, which is a common issue in publications including migrants [122]. In addition, very limited evidence is available comparing heat-related risks and prevention measures between MEM and non-MEM workers or MEM worker subgroups. Moreover, the results are not representative for the global situation, as 73.3% of the studies were conducted in North America (68.3% in the US), which only encompasses 22.1% of the global migrant worker population, while only 5% studies were conducted in (Northern, Southern and Western) Europe, where 24.2% of global migrant workers are living [14]. Although not all of these workers will be working outdoors and be exposed to occupational heat, it is clear that there is a lack of studies from Europe. Only studies published in English and Spanish were including, therefore publications in national languages were possibly missed. The potential publication bias was partially limited by including a grey literature search which identified one additional paper [63].

The present scoping review allowed to determine the body of the evidence about heat-related impacts, vulnerability, perception, behaviours and healthcare access in MEM outdoor workers identifying main themes and literature gaps. Further research is needed, including original studies assessing HRI prevalence in countries outside of the US, but also to explore whether MEM outdoor workers are at increased risk of adverse heat-related health outcomes compared to native outdoor workers and about the MEM specific risk-factors for heat-related health outcomes and access to healthcare after occupational heat exposure.

## Conclusion

MEM outdoor workers are at risk for high heat-related vulnerability, paralleled by barriers to heat prevention measures and some barriers to access to healthcare. These factors could be related to limited levels of HRI training, limited risk perception and adverse incentives to work faster, such as being paid at piece rate and fear of supervisors. In the context of climate change and rising global temperatures, there is a need to strengthen prevention efforts, including workplace organizational changes((e.g., regular breaks, reducing workload during hot weather) to improve safety and reduce heat workload as well as improving relationships and trust between MEM workers and their employers to be able reduce the burden of heat in this population.

## Key References

- El Khayat M, Halwani DA, Hneiny L, Alameddine I, Haidar MA, Habib RR. Impacts of Climate Change and Heat Stress on Farmworkers’ Health: A Scoping Review. Front Public Health. 2022;10:1–19**.**
  o This review shows that migrant farmworkers experience high levels of HRI symptoms.
- Ioannou LG, Testa DJ, Tsoutsoubi L, Mantzios K, Gkikas G, Agaliotis G, et al. Migrants from Low-Income Countries have Higher Heat-Health Risk Profiles Compared to Native Workers in Agriculture. J Immigr Minor Health. 2023;25:816–23.
  o This study from Cyprus shows that migrant agricultural workers originating from Low and Middle income countries (LMIC) experience higher levels of occupational heat strain, as compared to migrant workers from Upper-Middle Income countries (UMIC) and native workers from High Income Countries (HIC).
- Fleischer NL, Tiesman HM, Sumitani J, Mize T, Amarnath KK, Bayakly AR, et al. Public Health Impact of Heat-Related Illness Among Migrant Farmworkers. Am J Prev Med. 2013;44:199–206.
  o US study showing that internal migrant farmworkers experienced high levels of HRI symptoms and faced substantial barriers to preventing these symptoms.
- Mutic AD, Mix JM, Elon L, Mutic NJ, Economos J, Flocks J, et al. Classification of Heat-Related Illness Symptoms Among Florida Farmworkers. J Nurs Scholarsh Off Publ Sigma Theta Tau Int Honor Soc Nurs. 2018;50:74–82.
  o US study showing that international migrant farmworkers in the US reported a high burden of HRI symptoms.
- Arcury TA, Arnold TJ, Quandt SA, Chen H, Kearney GD, Sandberg JC, et al. Health and Occupational Injury Experienced by Latinx Child Farmworkers in North Carolina, USA. Int J Env Res Public Health. 2020;17.
  o One of the publications from the The Hired Child Farmworker Study providing evidence on heat-related illness and risk factors among child migrant farmworkers in the US.

## Supplementary Information

Below is the link to the electronic supplementary material.

Supplementary file1 (DOCX 32 KB)

## Data Availability

No datasets were generated or analysed during the current study.
