# Supplementary material for: Occupational Heat Stress Among Migrant and Ethnic Minority Outdoor Workers: A Scoping Review
Source: Curr Environ Health Rep. 2025 Mar 24;12(1):16. doi: 10.1007/s40572-025-00481-y (PMC11930879; doi:10.1007/s40572-025-00481-y)
Supplement: Supplementary file 1 — Supplementary file1 (DOCX 32 KB) [file 40572_2025_481_MOESM1_ESM.docx]

**Supplementary materials

Supplementary table 1: full search strategy**

| **#** | **Searches** |
| --- | --- |
| 1 | “heat*”[Title/Abstract] OR “sunstroke”[Title/Abstract] OR “sun stroke” [Title/Abstract:~3] OR “hot weather”[Title/Abstract:~3] OR “hot temperature”[Title/Abstract] OR “hot temperatures”[Title/Abstract:~3] OR “hot climate”[Title/Abstract:~3] OR “hot climates”[Title/Abstract:~3] OR “warm weather”[Title/Abstract:~3] OR “warm temperature”[Title/Abstract:~3] OR “warm temperatures”[Title/Abstract:~3] OR “warm climate”[Title/Abstract:~3] OR “warm climates”[Title/Abstract:~3] OR “WBGT”[Title/Abstract] OR “wet bulb globe temperature”[Title/Abstract] OR “extreme temperature”[Title/Abstract:~3] OR “extreme temperatures”[Title/Abstract:~3] OR “thermal discomfort” [Title/Abstract:~3] OR “temperature* change*”[Title/Abstract] OR “temperature* increase*” [Title/Abstract] OR "Extreme Hot Weather"[Mesh] OR "Heat Stress Disorders"[Mesh] OR "Hot Temperature"[Mesh] |
| 2 | “employ*”[Title/Abstract] OR “labour*”[Title/Abstract] OR “labor*”[Title/Abstract] or “occupation*”[Title/Abstract] or “job”[Title/Abstract] OR “jobs”[Title/Abstract] OR “work*”[Title/Abstract] OR “staff*”[Title/Abstract] OR “farmer*”[Title/Abstract] OR “farmwork*”[Title/Abstract] OR "Occupational Groups"[Mesh] OR "Occupational Exposure"[Mesh] OR "Occupational Health"[Mesh] |
| 3 | “Migra*”[Title/Abstract] OR “immigr*”[Title/Abstract] OR “emigr*”[Title/Abstract] OR “refugee*”[Title/Abstract] OR “asylum*”[Title/Abstract] OR “foreign*”[Title/Abstract] OR “undocument*”[Title/Abstract] OR “non-citizen*”[Title/Abstract] OR “citizenship*”[Title/Abstract] OR “transient worker”[Title/Abstract:~5] OR “displac*”[Title/Abstract] OR “diaspora*”[Title/Abstract] OR “latin*”[Title/Abstract] OR “hispanic*”[Title/Abstract] OR “expat*”[Title/Abstract] OR “nationalit*”[Title/Abstract] OR “non-resident”[Title/Abstract] OR “non-residents”[Title/Abstract] OR “newly-arrived” [Title/Abstract] OR “newcomer*”[Title/Abstract] OR “new-comer*”[Title/Abstract] OR “foreign-born”[Title/Abstract] OR “country origin”[Title/Abstract:~3] OR “country birth”[Title/Abstract:~3] OR “place birth”[Title/Abstract:~3] OR “birthplace”[Title/Abstract] OR “refugi*”[Title/Abstract] OR “asile”[Title/Abstract] OR “etrang*”[Title/Abstract] OR “sans-papiers” [Title/Abstract] OR “transitoire*”[Title/Abstract] OR “citoyen*”[Title/Abstract] OR “Transients and Migrants”[Mesh] OR “Refugees”[Mesh] OR “Emigrants and Immigrants”[Mesh]  OR “Ethnic and Racial Minorities"[MeSH] OR "Ethnicity"[MeSH] OR “Indigenous Peoples”[MeSH] OR “Minority Groups”[Mesh] OR “ethnic*”[Title/Abstract] OR “cultural divers*”[Title/Abstract] OR “cross-cultur*”[Title/Abstract] OR “cultural characteristics”[Title/Abstract:~3] OR “cultural characteristic”[Title/Abstract:~3] OR “cultural diversity”[Title/Abstract:~3] OR “culturally diverse”[Title/Abstract:~3] OR “linguistic diversity”[Title/Abstract:~3] OR “linguistically diverse”[Title/Abstract:~3] OR “CALD”[Title/Abstract] OR “minority group*”[Title/Abstract] OR “racial*”[Title/Abstract] OR “race*”[Title/Abstract] OR “Indigenous”[ Title/Abstract] |
| 4 | #1 AND #2 AND #3 |

**Supplementary table 2: qualitative results not presented in the main text**

| **Heat related knowledge and perception** | |
| --- | --- |
| Perception of heat | In Florida and North Carolina, US, several farmworkers mentioned extreme heat as a problem.[81,100] It was described as “*the hardest thing about working in agriculture*” and “*really, really, really bad*”.[42,100] |
| Knowledge and perception regarding symptoms and consequences of heat | Workers were able to correctly mention several HRI symptoms like dizziness, nausea, headache, muscle cramps, weakness and heart problems. They also associated turning pale, irritated eyes, stomach pain, heartburn, nose bleeds, flu, diarrhea, and pregnancy with HRI.[51,72] One study noted that women would mention less severe symptoms like dizziness, cramps and vomiting, while men would mention more severe symptoms such as fainting or falling.[51] Pregnant women thought the heat could cause a fetus to become agitated, resulting in increased fetal movement and possibly increased fetal heartbeats. They also said the fetus could get dehydrated when women do not drink enough and that it can “drown from heat”. One woman illustrated this by an example of a pregnant woman who´s unborn baby died throughout the working day.[55] |
| Knowledge and perception regarding risk-factors for and prevention of HRI | Workers in the US could mention several causes for HRI, including working long hours in the heat, not taking breaks in the shade or not drinking water, consuming drinks with much caffeine and alcohol, working quickly, not being well acclimatized and wearing dark clothing.[66,84] Personal factors mentioned included pre-existing chronic health conditions, being overweight, lack of general fitness or sleep, poor diet and taking medications.[66,72] Regarding heat-mitigation strategies they mentioned drinking water and electrolyte beverages, taking breaks and dressing appropriately, including long‐ sleeved shirts, long pants and gloves and wearing a hat.[39,55,84] Some pregnant women, working under a black shade cloth, said “*there was no way to protect you from that intense heat*.”[55] |
| Knowledge and perception regarding treatment HRI | When suffering from HRI, workers mentioned to stop working and sit down in the shade. In case someone else suffers from HRI, workers knew they need to remove excess layers and loosen tight clothing, provide fluids, check the pulse, get help from supervisors or other co-worker and call an ambulance.[66] There was disagreement on cooling with ice, as some considered it useful while others thought it might cause headaches and fainting.[66] Other suggestions were strong coffee without sugar and in case someone was unconscious, waking them up by smelling onions or alcohol.[66] In one study, participants talked about using over-the-counter medicines such as Tylenol or XL-3 (cough medicine) to treat headaches and nausea.[72] |
| Relationship with supervisors | Some minors said they think the employers cared about their safety while others said that they just care about the work being finished.[39] Several adult workers reported not thinking their companies and supervisors cared about their safety.[84] A minor said that one supervisor would make everyone stop working when it would get too hot while another one would leave it up to the workers themselves to decide.[39] Adult workers mentioned being afraid to speak up to superiors about workplace conditions as individuals or groups would be fired if they complained or didn’t work hard: *“You can get heat exhaustion, but you don’t inform [a supervisor]. You can deal with it because they’re going to fire you or because you need work”.*[50,81] |
| **Fluid intake** | |
| Water consumption | At several sites workers mentioned not liking the water provided by employers for various reasons including taste, appearance, odor, temperature and being located next to the toilets.[41,50,51,66,72,81] Additional reasons for not drinking water were not wanting to interrupt work to drink or go to the toilet and possibly upset the supervisor or make less money, especially when working at piece rate, and not bringing bottles into the fields to prevent contaminating the water with chemicals.[55,66,81,100] Finally, workers in various studies mentioned a belief that drinking cold water can make you sick, especially when overheated, with symptoms including nausea and vomiting, muscle spasms, cramps in the lungs, pneumonia, palpitations or even heart attacks.[40,51,55,72] Therefore, some adults reported sometimes drinking beers to overcome thirst and minors mentioned drinking sodas for this reason.[40,51,66,72] |
| Cooling | Workers who suffered from HRI symptoms reported resting and drinking beverages with electrolytes. Workers also reported wetting their bandanas, hats or head to help cool down.[72] Several workers mentioned that cooling the body down too quickly, for example by drinking very cold water, could be dangerous: “*When the temperature in your body is very hot then you throw something very cold on it, it breaks*”. Therefore, they said you should not drink too much water at once.[51] |
| **Breaks and cooling** | |
| Breaks | Several minors said they could work slower, take breaks or leave early when it was really hot, in most cases these minors were younger and working with family members.[41,42] Some others had examples of being screamed at and threatened to be replaced when working too slow or not being allowed breaks: “*Well, if they’re rushing you, then you will feel stressed out. It’s like, you need a break. Or you’ll pass out or something*.”[41,42] Examples were mentioned of workers not being allowed to take planned breaks before the task was done or not giving the crew a lunch break at all and supervisors threatening to take away an hour of salary if taking a break or slowing down.[41,42] |
| Working at piece rate | Piece work was mentioned as a reason to keep working “*until we can’t handle it anymore*” and taking few breaks: “*When we see that we have only earned 50 or 40, we have to continue to reach at least 100, and then we take it easy*”.[51,72] Workers said they would “abuse” or “hurt” their bodies to fill more buckets and make more money.[72,81] Supervisors encouraging piece workers to drink water and take breaks were perceived as a drag on the potential to earn wages.[51] On the other hand, workers with hourly payment often portrayed employers as being disinterested in workers´ wellbeing and make them working long hours so they would end up exhausted.[51,72] |
| **Clothing** | |
| Clothing | Workers in the US explained that sometimes being covered up could increase the heat or cause HRI but that they would do it to prevent adverse health consequences from chemicals.[66] Likewise, Some workers would wear denim jeans because “*they are* *thicker and won’t get caught on tree branches*.”[66] In addition, some participants would purposely wear dark clothes or girdles for back support, despite knowing it increases heat, because they would sweat more and believed it could help them lose weight.[66] Some workers indicated that it was important to use clothing to protect them from the sun, while another mentioned taking breaks in the shade was sufficient as they were all “*morenos*” (brown).[72] |
